# Cesarean delivery on maternal request and its influencing factors in Chongqing, China

**DOI:** 10.1186/s12884-021-03866-7

**Published:** 2021-05-19

**Authors:** Ruibin Deng, Xian Tang, Jiaxiu Liu, Yuwen Gao, Xiaoni Zhong

**Affiliations:** 1 School of Public Health and Management, Chongqing Medical University, #1 Yixue Rd, 400016 Chongqing, China; 2 Research Center for Medicine and Social Development, Chongqing Medical University, Chongqing, China; 3 Innovation Center for Social Risk Governance in Health, Chongqing Medical University, 400016 Chongqing, China

**Keywords:** Cesarean delivery rate, Surgical indications, Cesarean delivery on maternal request, Influencing factors, China

## Abstract

**Background:**

A high rate of cesarean delivery has become a cause of global concern. Although the rate of cesarean delivery has declined over recent years, it remains at a high level largely because of cesarean delivery on maternal request (CDMR). Unnecessary cesarean delivery has limited significance in benefiting maternal and infant physical health; in some ways, it might pose potential risks instead. With the implementation of the “Two-child Policy” in China, an increasing number of women plan to have a second child. Accordingly, how to handle the CDMR rate in China remains an important issue.

**Methods:**

Data were collected from a longitudinal follow-up study conducted in Chongqing, China, from 2018 to 2019. A structured questionnaire was administered to subjects for data collection. Basic information, including demographic characteristics, living habits, medical history, and follow-up data of pregnant women, as well as their families and society, was collected. Additionally, delivery outcomes were recorded. Logistic regression was performed to analyze the factors influencing CDMR.

**Results:**

The rate of cesarean delivery in Chongqing, China was 36.01 %, and the CDMR rate was 8.42 %. Maternal request (23.38 %), fetal distress (22.73 %), and pregnancy complications (9.96 %) were the top three indications for cesarean delivery. Logistic regression analysis showed that older age (OR = 4.292, 95 % CI: 1.984–9.283) and being a primiparous woman (OR = 6.792, 95 % CI: 3.230-14.281) were risk factors for CDMR. In addition, CDMR was also associated with factors such as the tendency to choose cesarean delivery during late pregnancy (OR = 5.525, 95 % CI: 2.116–14.431), frequent contact with mothers who had undergone vaginal deliveries (OR = 0.547, 95 % CI: 0.311–0.961), and the recommendation of cesarean delivery by doctors (OR = 4.071, 95 % CI: 1.007–16.455).

**Conclusions:**

“Maternal request” has become the primary indication for cesarean delivery. The occurrence of CDMR is related to both the personal factors of women during pregnancy and others. Medical institutions and obstetricians should continue popularizing delivery knowledge among pregnant women, enhancing their own professional knowledge about delivery, adhering to the standard indications for cesarean delivery, and providing pregnant women with adequate opportunities for attempting vaginal delivery.

**Supplementary Information:**

The online version contains supplementary material available at 10.1186/s12884-021-03866-7.

## Background

Cesarean delivery is an obstetric operation used to address dystocia and high-risk pregnancy. Medically necessary cesarean delivery plays an essential role in protecting the lives of high-risk pregnant women and newborns. However, as cesarean delivery is currently performed more frequently than before, its adverse effects on mothers and infants have gradually been revealed. Compared with those who undergo vaginal delivery, women who have a cesarean delivery are more prone to febrile diseases [1] and small bowel obstruction [2], and they have a higher risk of severe acute maternal morbidity (SAMM) [3], postpartum depression [4], and postpartum death [5]. Moreover, infants born via cesarean section might be subjected to subtle physiological modulation due to hormonal, physiological, bacterial and medical interventions [3]; they are more susceptible to respiratory and immune system diseases [6–9]; and they may even sustain a lasting impact on their growth and development [10, 11]. Nevertheless, there remain numerous misunderstandings about cesarean delivery among pregnant women [12], resulting in a popular demand for cesarean delivery.

Cesarean delivery on maternal request (CDMR) is also called cesarean delivery with social factors and cesarean delivery without medical indications. In 2007, the American College of Obstetricians and Gynecologists (ACOG) defined CDMR as a primary cesarean delivery on maternal request in the absence of any medical or obstetric indication [13]. CDMR has limited significance in benefiting maternal and infantile physical health in contrast to cesarean delivery with medical indications. Instead, the cesarean delivery itself poses potential risks to pregnant women and infants when performed without a medical need. For instance, mothers are more likely to suffer from short-term ill effects, such as wound infection [14, 15]. Additionally, children born via CDMR are at an elevated risk of emotional and behavioral problems while they are under school age [16]. In addition, CDMR prolongs hospitalization, leading to a waste of medical resources and an increase in the hospitalization cost to pregnant women [17]. Therefore, unnecessary cesarean delivery in the absence of any medical or obstetric indications should be strictly avoided.

The high rate of cesarean delivery has always been a hot issue and has caused international concern [18–20]. The global cesarean delivery rate was 21.1 % in 2015, almost twofold of that in 2000 [21]. According to a World Health Organization (WHO) survey in 2010, the average cesarean delivery rate in nine countries in Asia was 27.3 %, whereas it reached 46.2 % in China [22]. With the intervention of the Chinese government in terms of the health institution reformation and other aspects, the rate of cesarean delivery has shown a downward trend in recent years [23, 24]. In 2018, the rate of cesarean delivery in China was 36.7 % [25]. Although lower than before, this rate is still far higher than the 10-15 % recommended by the WHO. As the economy is developing, the indications for cesarean delivery have changed, and CDMR has made a major contribution to the increase in the rate of cesarean delivery [21, 26–28]. The population of CDMR reported internationally accounts for 4.4-17.3 % of the total cesarean delivery population [22, 29–31], whereas that number is 25.2-31.4 % in China [22, 26, 32], which is far higher than the international average level. With the implementation of the “Two-child Policy” in China, an increasing number of women plan to have a second child, especially among older mothers who show a stronger preference for CDMR than younger mothers [33, 34]. Globally, how to control the rate of CDMR in China is still an important issue.

Until now, few reports have analyzed the rate of cesarean delivery in southwestern China. Among those that do exist, their publication dates are relatively old. Only a small number of reports have covered CDMR and its influencing factors. Our analyses were based on a longitudinal follow-up observation study conducted in Chongqing (a municipality in southwestern China) from 2018 to 2019 and are expected to depict the pattern of the delivery mode of pregnant women after the implementation of the “Two-child Policy”. Grounded in these data, we explored the influencing factors for the occurrence of CDMR in terms of pregnant women, as well as their families and society, to provide theoretical support for reducing the rate of unnecessary cesarean delivery without medical indications.

## Methods

### Data sources

The data in this study came from the “Study on the Public Opinion Propagation Model for Generative Mechanism and Regularity of Cesarean Delivery Behavior” (Project No. 71,573,027) initiated by the National Natural Science Foundation of China. This research was carried out in Chongqing, China, and participants in four hospitals, two of which were in economically wealthy regions and two of which were in economically poor regions, were recruited. This study was approved by the Ethics Committee of Chongqing Medical University.

### Participants

All of the pregnant women who participated in their first pregnancy examination in one of the abovementioned four hospitals from January 2018 to September 2018 and planned to give birth in the hospital were screened. Participants who met the exclusion criteria (women with a history of cesarean delivery or with health problems, such as a mental illness) were excluded, and the remaining participants who met the inclusion criteria (women with a singleton pregnancy, gestational age < 15 weeks, signed informed consent and willingness to make follow-up arrangements) were included in our study. The demographic characteristics and prenatal examination information of the included pregnant women were then obtained.

### Study content and measurements

This study is based on the statistics obtained by structured questionnaires. An additional file presents the English version of our original Chinese questionnaires (see Additional file 1). The variables in the questionnaire were developed on the basis of published reports [33, 35, 36], prenatal examination reports and healthcare reports of the participants and were jointly evaluated and modified by public health experts, obstetricians and psychologists. Three aspects were involved, including basic information and personal factors, family factors and social factors. A follow-up survey was conducted in early pregnancy (< 15 weeks), middle pregnancy (15 weeks-27 weeks and 6 days) and late pregnancy (28 weeks–before delivery). This study mainly used the follow-up data of the late pregnancy period closest to the delivery time as the basic situation of the pregnant women before delivery. After delivery, the delivery situation was recorded, and the delivery outcome was obtained.

#### Basic information and personal factors

The content of the investigation included demographic characteristics (age, residency, level of education, occupation and monthly per capita household income), prenatal examination information (height, weight, history of pregnancy, previous medical history), personal behaviors (drinking history, smoking history and exercise habits) and psychological condition (stress, anxiety and depression).

The psychological condition was described by standard scales. Stress was measured by the Pregnancy Pressure Scale (PPS) compiled by Zhanghui Chen et al. [37]. The PPS comprises 30 items, and the average score calculated from the total score of all questions is used to measure the stress, with 0 for no stress and a score ≥ 0.01 for stress. The Cronbach’s α coefficient for PPS in this study was 0.949, the KMO test statistic was 0.954, and Bartlett’s spherical test showed statistical significance (*χ*2 = 13041.917, *P* < 0.001).

Anxiety was measured using the Hamilton Anxiety Scale (HAMA) [37], which contains two dimensions, mental anxiety and somatic anxiety, with a total of 14 items, and the total score of all of the questions was calculated. A score of ≤ 7 indicates no anxiety, a score of 8–14 indicates suspected anxiety, and a score of ≥ 15 indicates anxiety. The Cronbach’s α coefficient of HAMA in this study was 0.919, the KMO test statistic was 0.942, and Bartlett’s spherical test showed statistical significance (*χ*2 = 5071.247, *P* < 0.001).

Depression was measured by the Self-rating Depression Scale (SDS), which calculated a depression severity index (actual total score/highest possible score for all entries) [38]. An index of < 0.5 indicates no depression, and ≥ 0.5 indicates depression. The Cronbach’s α coefficient for SDS in this study was 0.817, the KMO test statistic was 0.888, and Bartlett’s spherical test showed statistical significance (*χ*2 = 1918.036, *P* < 0.001).

#### Family factors

Family factors mainly included the families’ advice on delivery mode and family care. The family adaptation partnership growth affection (APGAR) was used to measure the degree of family care [39]. The APGAR uses a total score of entries to assess maternal satisfaction with family functions. A score of 0–3 represents severe impairment in family functions, a score of 4–6 represents moderate impairment in family functions, and a score of 7–10 represents good family functions. The Cronbach’s α coefficient of APGAR in this study was 0.848, the KMO test statistic was 0.827, and Bartlett’s spherical test showed statistical significance (χ2 = 1457.359, *P* < 0.001).

#### Social factors

Social factors included social support, doctors’ and friends’ advice on delivery mode, and the delivery mode of the surrounding mothers and medical staff service. Social support was measured by the Social Support Rating Scale (SSRS) complied by Xiao Water in 1986 [40]. A total score of all entries was calculated, with a score < 35 for a low level of social support, 35–45 for a medium level, and > 45 for a high level. Cronbach’s α coefficient of the SSRS in this study was 0.694, the KMO test statistic was 0.818, and Bartlett’s spherical test showed statistical significance (χ2 = 1084.008, *p* < 0.001).

### Statistical analysis

The database was established using EpiData 3.1 software (EpiData Associations, Odense, Denmark), and real-time double entry and logical verification of the data were carried out. Statistical analysis was performed by SAS 9.4 software (SAS Institute, Cary, NC, USA). In data processing, some continuous variables (age, family care score, social support score, pregnancy stress score, anxiety score and depression score) were converted into categorical variables. According to the purpose of this study, women without clinical medical indications during delivery were analyzed, whereas those with operative vaginal delivery (OVD) and those with cesarean delivery with medical indications (in this study, the indications for cesarean delivery surgery were mainly the first indication). Except for CDMR, in this study, all indications were designated medical indications (Table 1), and those who did not complete the late pregnancy follow-up were excluded. The *χ2* test and Fisher’s exact test were used to compare the differences in delivery modes among women with different characteristics and to screen for initial potential variables. Then, the variables with *P* < 0.1 in univariate analysis and/or with significance from a professional perspective were included in the multivariate logistic stepwise regression model to obtain the factors influencing CDMR. A few variables were missing, and observations with missing values (all of which were less than 1.5 %) were excluded from the regression analysis.

**Table 1:** Indications for cesarean delivery

Indications for cesarean delivery	Frequency	Percentage
	**(*N* = 462)**	**(%)**
Cesarean delivery on maternal request (CDMR)	108	23.38
Fetal distress	105	22.73
Pregnancy complications	46	9.96
Abnormal amniotic fluid	43	9.31
Fetal position abnormality	35	7.58
Cephalopelvic disproportion	35	7.58
Scarred uterus	19	4.11
Placenta previa	17	3.68
Premature rupture of membranes	11	2.38
Fetal macrosomia	10	2.16
Others	33	7.14

Pregnancy complications include pregnancy hypertension, intrahepatic cholestasis of pregnancy (ICP), and gestational diabetes mellitus (GDM). Scarred uterus includes uterine fibroids and cervical scars after excision

## Results

### Basic information

#### Selection of participants

**Fig. 1:**
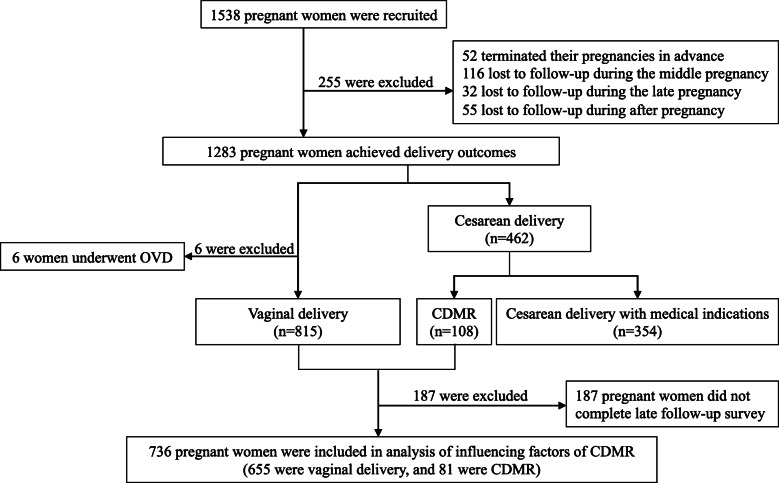
Flow chart of participant categorization and disposition

A total of 1538 pregnant women from four medical institutions were recruited for this study. By July 2019, the delivery outcomes of 1283 pregnant women were obtained. The factors influencing CDMR were analyzed by taking 736 women who had no clinical medical indication during delivery as the study subjects. The selection of the research subjects is shown in Fig. 1.

#### Delivery situation and the indications of cesarean delivery

Among the 1283 pregnant women, 815 had a vaginal delivery (i.e., spontaneous vaginal delivery without a medical intervention), 6 had an OVD (excluded from the analysis), and 462 had a cesarean delivery (of which 108 were CDMR), with a cesarean delivery rate of 36.01 %. “Maternal request” was the primary indication for cesarean delivery, accounting for 23.38 % (108/462) of the total number of cesarean deliveries. Additionally, the overall CDMR rate was 8.42 % (108/1283). In addition, the top five medical indications were fetal distress, pregnancy complications, abnormal amniotic fluid, a fetal position abnormality and cephalopelvic disproportion (Table 1).

### General characteristics

The age of the participants ranged from 16 years to 44 years, and 62.09 % of them were primiparous women. Among them, 62.50 % lived in urban areas, and 37.36 % lived in rural areas. The overall level of education was relatively high, as 40.90 % of the women were highly educated. Moreover, 40.63 % of them had a monthly per capita household income between 3,000 and 5,000 RMB. Women with a family monthly income of more than 5,000 RMB accounted for 35.05 % of the sample, whereas those below 3,000 RMB accounted for 24.05 %. Nearly half of the women (48.78 %) were housewives or unemployed, and 82.88 % of them exercised during pregnancy. In addition, most of the pregnant women (87.50 %) had stress symptoms in late pregnancy, and the frequencies of anxiety and depression symptoms were 10.60 and 2.85 %, respectively (Table 2).

**Table 2:** Sample characteristics and univariate analysis of pregnant women’s data

Characteristics	Total		Vaginal delivery		CDMR		*P* value
	**n**	**%**	**n**	**%**	**n**	**%**	
Total	736	100.00	655	89.00	81	11.00	
**Demographic and socioeconomic characteristics**							
Age^a^							0.1823
<25	307	41.71	277	90.23	30	9.77	
25–30	324	44.02	290	89.51	34	10.49	
>30	98	13.32	82	83.67	16	16.33	
Place of residence^a^							0.0753
Urban	460	62.50	402	87.39	58	12.61	
Rural	275	37.36	252	91.64	23	8.36	
Have you ever received higher education^a^							0.0623
No	433	58.83	393	90.76	40	9.24	
Yes	301	40.90	260	86.38	41	13.62	
Occupation^a^							0.4011
Employed	376	51.09	331	88.03	45	11.97	
Housewife/Unemployed	359	48.78	323	89.97	36	10.03	
Monthly per capita household income (RMB)^a^							0.0602
≤ 3000	177	24.05	160	90.40	17	9.60	
3001–5000	299	40.63	273	91.30	26	8.70	
≥ 5001	258	35.05	220	85.27	38	14.73	
Payment method of medical expenses^a^							0.5926
At one’s own expense	292	39.67	262	89.73	30	10.27	
Medical insurance	442	60.05	391	88.46	51	11.54	
** Prenatal information**							
BMI^a^							0.5312
< 18.5	131	17.80	118	90.08	13	9.92	
18.5–23.9	511	69.43	456	89.24	55	10.76	
≥ 24	90	12.23	77	85.56	13	14.44	
Drinking history							0.2168^*^
Yes	8	1.09	6	75.00	2	25.00	
No	728	98.91	649	89.15	79	10.85	
Smoking history							1.0000^*^
Active smoking	8	1.09	7	87.50	1	12.50	
Passive smoking	18	2.45	16	88.89	2	11.11	
No smoking	710	96.47	632	89.01	78	10.99	
Parity							**< 0.0001**
0 (primiparity)	457	62.09	388	84.90	69	15.10	
≥ 1	279	37.91	267	95.70	12	4.30	
Previous Medical History							0.2424
Yes	50	6.79	42	84.00	8	16.00	
No	686	93.21	613	89.36	73	10.64	
Number of Abortions							0.5282
0	450	61.14	404	89.78	46	10.22	
1	171	23.23	152	88.89	19	11.11	
≥ 2	115	15.63	99	86.09	16	13.91	
**Personal factors**							
Willingness of delivery mode in late pregnancy							**< 0.0001**
Uncertain	295	40.08	265	89.83	30	10.17	
Tending to choose vaginal delivery	409	55.57	371	90.71	38	9.29	
Tending to choose cesarean delivery	32	4.35	19	59.38	13	40.63	
Exercise during pregnancy							0.3272
Yes	610	82.88	546	89.51	64	10.49	
No	126	17.12	109	86.51	17	13.49	
Attending a school for pregnant women during pregnancy							0.9026
Yes	350	47.55	312	89.14	38	10.86	
No	386	52.45	343	88.86	43	11.14	
Late-pregnancy stress^a^							0.6195
No	84	11.41	76	90.48	8	9.52	
Yes	644	87.50	571	88.66	73	11.34	
Late-pregnancy anxiety^a^							0.3818
No	648	88.04	578	89.20	70	10.80	
Yes	78	10.60	67	85.90	11	14.10	
Late-pregnancy depression^a^							0.1539^*^
No	711	96.60	630	88.61	81	11.39	
Yes	21	2.85	21	100.00	0	0.00	
**Family factors**							
Family care^a^							0.8520
Low	26	3.53	24	92.31	2	7.69	
Medium	174	23.64	155	89.08	19	10.92	
High	534	72.55	474	88.76	60	11.24	
Husband’s advice							0.0315^*^
Does not recommend cesarean delivery	707	96.06	633	89.53	74	10.47	
Recommends cesarean delivery	29	3.94	22	75.86	7	24.14	
Parents’ advice							0.0033^*^
Do not recommend cesarean delivery	721	97.96	646	89.60	75	10.40	
Recommend cesarean delivery	15	2.04	9	60.00	6	40.00	
In-laws’ advice							0.0061^*^
Do not recommend cesarean delivery	724	98.37	648	89.50	76	10.50	
Recommend cesarean delivery	12	1.63	7	58.33	5	41.67	
**Social factors**							
Social support^a^							0.7792
Low	153	20.79	138	90.20	15	9.80	
Moderate	431	58.56	382	88.63	49	11.37	
High	129	17.53	113	87.60	16	12.40	
Delivery mode of surrounding mothers							**0.0268**
Mainly vaginal delivery	347	47.15	320	92.22	27	7.78	
Mainly cesarean delivery	105	14.27	89	84.76	16	15.24	
The two delivery methods are equal	284	38.59	246	86.62	38	13.38	
Friends’ advice							0.0321^*^
Do not recommend cesarean delivery	713	96.88	638	89.48	75	10.52	
Recommend cesarean delivery	23	3.13	17	73.91	6	26.09	
Doctors’ advice^a^							0.0340^*^
Do not recommend cesarean delivery	714	97.01	638	89.36	76	10.64	
Recommend cesarean delivery	12	1.63	8	66.67	4	33.33	
Medical staff service							0.6003^*^
Good	693	94.16	618	89.18	75	10.82	
General	40	5.43	34	85.00	6	15.00	
Poor	3	0.41	3	100.00	0	0.00	

^*^ Fisher’s exact probability test

^a^indicates loss of data

Bold value indicates statistical significance at *P* < 0.05

### Willingness of delivery mode

In late pregnancy, 55.51 % of the women preferred vaginal delivery, 4.46 % of them preferred cesarean delivery, and 40.03 % of them did not have a clear choice of delivery mode (Table 2). Inquiries about the tendency toward delivery modes were conducted in late pregnancy. Pregnant women (*N* = 327) who had no definite willingness to undergo cesarean delivery or who were willing to have a cesarean delivery were asked about their motivations to have a cesarean delivery, whereas those who were willing to have a vaginal delivery were asked about their motivations to have a vaginal delivery (data not shown). Some women might have had multiple motivations to choose a cesarean delivery at the same time. Most (55.35 %) women were afraid of vaginal labor pains and believed that cesarean delivery caused less pain. In addition, the doctors’ advice, lack of confidence in vaginal delivery and a belief that cesarean delivery was safer for children were the main motivations for women to choose a cesarean delivery (Table 3).

**Table 3:** The motivations for cesarean delivery

Motivations	Frequency	Percentage
	**(*N* = 327)**	**(%)**
Labor pain is less than with vaginal delivery, afraid of vaginal labor pain	181	55.35
The doctor suggested that cesarean delivery was necessary	128	39.14
No confidence in vaginal delivery, fear of failure of vaginal delivery before performing cesarean delivery	89	27.22
Safer for children	68	20.80
It is safe to the mother and saves time and effort	60	18.35
Faster body shape recovery	53	16.21
Protection of perineal tissue, does not affect postpartum sexual life	40	12.23
Kids are smarter and healthier	35	10.70
You can choose a good day	25	7.65
Fear of pregnancy complications, such as uterine rupture	19	5.81
Suggestions from family and friends	16	4.89
Precious children, such as test tube babies, years of infertility	3	0.92

### Multivariate analysis of the factors influencing CDMR

In the univariate analysis, parity, willingness to deliver in late pregnancy, delivery mode of the surrounding mothers, husbands’ advice on delivery mode, parents’ advice on delivery mode, in-laws’ advice on delivery mode, friends’ advice on delivery mode, and doctors’ advice on delivery mode were all associated with different delivery modes (*P* < 0.05) (Table 2).

A logistic stepwise regression model was used in the multivariate analysis. The mode of delivery was taken as the dependent variable (0 = vaginal delivery, 1 = CDMR), whereas variables in the univariate analysis (*P* < 0.1) were selected as independent variables. Combined with information from the literature and professional knowledge, maternal age and BMI were included in the model for adjustment [33, 35]. As shown in Table 4, compared with women under 25 years of age, women over 30 years of age were 4.3 times more likely to have CDMR (OR = 4.292, 95 % CI: 1.984–9.283). Compared with multiparous women, primiparous women had a higher risk of CDMR (OR = 6.792, 95 % CI: 3.230-14.281). Moreover, women who tended to choose cesarean delivery before birth were 5.5 times more likely to have CDMR than women with no clear intention (OR = 5.525, 95 % CI: 2.116–14.431). However, women in frequent contact with mothers who had undergone vaginal deliveries had a reduced risk of CDMR (OR = 0.547, 95 % CI: 0.311–0.961). In addition, pregnant women are more likely to choose cesarean delivery if their doctors recommended it (OR = 4.071, 95 % CI: 1.007–16.455).

**Table 4:** Multivariate logistic stepwise regression analysis (*N* = 713)

Independent variables	β	Wald	*P* value	OR (95 % CI)
Age				
<25				reference
25–30	0.3541	1.4986	0.2209	1.425(0.808–2.512)
>30	1.4566	13.6922	**0.0002**	4.292(1.984–9.283)
Parity				
≥ 1				reference
0 (primiparity)	1.9157	25.5250	**< 0.0001**	6.792(3.230-14.281)
Willingness to undergo delivery mode in late pregnancy				
Uncertain				reference
Tending to choose vaginal delivery	0.2184	0.5983	0.4392	1.244(0.715–2.164)
Tending to choose cesarean delivery	1.7094	12.1784	**0.0005**	5.525(2.116–14.431)
Delivery mode of surrounding mothers				
The two delivery methods are equal				reference
Mainly vaginal delivery	-0.6041	4.4012	**0.0359**	0.547(0.311–0.961)
Mainly cesarean delivery	-0.4176	1.2558	0.2624	0.659(0.317–1.367)
Doctors’ advice				
Do not recommend cesarean delivery				reference
Recommend cesarean delivery	1.4038	3.8800	**0.0489**	4.071(1.007–16.455)

*β* indicates the standardized regression coefficient. *OR* indicates the adjusted odds ratio. *95 % CI* indicates the 95 % confidence interval. The bold value indicates *P* < 0.05, with statistical significance

## Discussion

### Cesarean delivery rate and indications

In this study, the cesarean delivery rate in Chongqing, China, was 36.01 %. This statistic is consistent with the national overall cesarean delivery rate provided in the report on the development of China’s maternal and child health in 2019 [25], which is still at a high level compared to the global average [21]. The CDMR rate was 8.42 %, accounting for 23.88 % of the cesarean delivery population. This finding is similar to the results of some previous studies [24, 35], which suggested that the rates of cesarean delivery and CDMR in Chongqing are at the average level in China. Although the WHO no longer gives a recommended specific value of the cesarean delivery rate, it still warns against unnecessary cesarean delivery [41]. In recent years, China has taken some measures, such as health education, painless delivery and the introduction of corresponding policies to reduce the rate of cesarean delivery [42]. Therefore, the high cesarean delivery rate may be due to other factors.

This study found that “maternal request” was the most frequent indication for cesarean delivery, a finding that is in accordance with those of other studies [21, 26]. In recent decades, with the development of the social economy and the continuous improvement of medical technology, the indications for cesarean delivery have changed. Additionally, the main indications have changed from maternal or fetal physiological status to social psychological factors [27, 28]. At the beginning of its popularization, cesarean delivery was mainly used to solve various high-risk pregnancy problems, such as dystocia, which saved the lives of a large number of high-risk pregnant women and newborns. However, with the widespread use of cesarean delivery, some women have misunderstandings about delivery knowledge and think that cesarean delivery is the safest method of delivery [12]. A research report in the United States showed that nearly half of obstetricians believe that pregnant women have the right to choose cesarean delivery [43], and Chinese obstetricians have the same belief [36]. Chongqing Health Statistics Information Center reported in 2015 that cesarean delivery was earning preference of younger generations [44]. This evidence suggests that the public tends to believe that cesarean delivery is a delivery mode that can be chosen freely.

### Influencing factors

#### Personal factors

This study found that maternal age and number of deliveries were associated with the presence of CDMR. Older women (> 30 years old) had a higher risk of developing CDMR than younger women (< 25 years old). The same conclusion was reached in previous international studies [33, 34]; older women often had no plan for pregnancy and seldom worried about the risk of vaginal birth after cesarean section (VBAC) when choosing a delivery mode. At the same time, with increasing age, women have a higher risk of pregnancy complications and adverse pregnancy outcomes [45, 46]. Although the general definition of older mothers is women over 35 years old, some researchers have pointed out that the at-risk age associated with different adverse outcomes varies, and the risk of some adverse outcomes is still very high among some women under age 35, even as young as 30 years old [47]. Pregnant women will be more concerned about the risk of vaginal childbirth if they are in a high-risk physiological condition.

However, women who have experienced childbirth in the past have fewer concerns about vaginal delivery. Our study confirms this view that women who are giving birth for the first time are more likely to choose cesarean delivery, even without any medical indications, compared to women who have previously experienced delivery. According to a Norwegian study, some primiparous women planned to have cesarean delivery since they were teenagers because of their fear of childbirth and a lack of understanding of fertility [48]. In China, the “two-child policy” was implemented in 2016, allowing a couple to have two children. If a woman who is giving birth for the first time chooses cesarean delivery, a trial of labor after cesarean delivery (TOLAC) is less likely to be her preference when giving birth to the second child [49]. Therefore, the choice of delivery mode for primiparous women is very important to control the overall cesarean delivery rate.

In late pregnancy, pregnant women are preparing to give birth. During this period, most pregnant women have adapted to the pregnancy process and have a certain degree of understanding of delivery knowledge. Interestingly, in our study, nearly half of the women still had no definite intention relating to the mode of delivery, and a small number of them preferred cesarean delivery. Furthermore, the results of the regression analysis showed that compared to women who had no definite willingness to deliver using a certain mode, women who preferred cesarean delivery had a higher probability of a cesarean delivery during the actual delivery. This suggests that we need to strengthen the guidance offered to pregnant women in late pregnancy to provide them with correct understanding of their delivery knowledge and mode to reduce the unnecessary cesarean delivery rate through interventions near the prenatal period.

In addition, when exploring the motivation of pregnant women in late pregnancy to consider cesarean delivery, we found that “afraid of vaginal labor pains, think cesarean delivery is less painful” was the most important factor. Moreover, nearly one-third of women did not have confidence in vaginal delivery. Tocophobia is very common in pregnant women and is one of the important reasons for CDMR [50–52]. This study also revealed that some pregnant women think that cesarean delivery is “safer for children”, “it is safe to the mother and saves time and effort” and “kids are smarter and healthier”. These misunderstandings of delivery knowledge are also important reasons for pregnant women to consider cesarean delivery. In addition, consistent with some international studies [53, 54], pregnant women’s concerns about their own physiological condition, such as “faster body shape recovery”, “protection of perineal tissue, does not affect postpartum sexual life”, and “fear of pregnancy complications such as uterine rupture”, make them worry about vaginal delivery. Moreover, due to China’s unique social and cultural influence, some people in the country connect the birth time of a child with his or her fortune and want to give birth at a particular time for an “auspicious” future. We found that 7.65 % of women still considered that selecting a certain day for delivery as a “good day” was an adequate reason for a cesarean delivery.

#### Influence of others

In addition to a pregnant woman’s personal factors, other people around them also affected their choice of delivery mode. We found that if the main delivery mode of the surrounding mothers was vaginal delivery, the probability of cesarean delivery was reduced by half. The successful experience of other parturient women’s vaginal delivery increased their confidence in delivery, whereas a failure of a trial of labor in other women possibly made them fear vaginal delivery and choose cesarean delivery. Regarding the choice of delivery mode of pregnant women being affected by others, the current research has mainly focused on families and friends. There are few reports on the influence of surrounding mothers on pregnant women. It is suggested that medical institutions guide active communication among pregnant women and strengthen their understanding of correct delivery knowledge among pregnant women in the process of waiting for labor.

In addition, our study showed that doctors’ advice played a key role in the choice of delivery mode. Compared with those who did not recommend cesarean delivery, pregnant women whose doctors recommended cesarean delivery had a higher risk of choosing cesarean delivery. We also found that a doctor recommendation was an important motivation for pregnant women to consider cesarean delivery. Previous studies have reported that the cesarean delivery rate of different doctors within the same institution can vary up to threefold, but no difference in patient characteristics or short-term neonatal outcomes has been observed [55], which shows that doctors’ personal decision making has a significant impact on the cesarean delivery rate. It has also been shown in a study in Brazil that doctors’ opinions have major impacts on the decisions of pregnant women, especially those made about the delivery mode [56].

At the same time, some pregnant women rely on the advice of obstetric medical staff and believe that it is their responsibility to make a decision about the mode of delivery [57]. However, obstetricians tend to choose cesarean delivery to avoid lawsuits caused by unexpected complications during the process of vaginal delivery, and surgical delivery can simultaneously bring them more income [58]. A survey of obstetricians showed that those with lower educational backgrounds and less work experience who thought that the advantages of cesarean delivery outweighed the disadvantages had a higher incidence of CDMR among their patients [36]. Therefore, we suggest that obstetric medical staff should enhance their understanding of professional knowledge, adhere to the indications for cesarean delivery, and give pregnant women a full opportunity to undergo a trial of labor.

### Limitations

This study had several limitations. The participants were from only one province in China. Furthermore, because of the exclusion criteria, no women had a history of cesarean delivery. Therefore, the cesarean delivery rate in this study is not representative of all Chinese mainland pregnant women. In addition, the main variables only captured information about the group and cross-sectional data from late pregnancy, which might not fully reflect the impact of the entire pregnancy status on the delivery outcome. Therefore, in a follow-up study, longitudinal data from multiple follow-up should be considered for analysis.

## Conclusions

CDMR has become the primary indication for cesarean delivery in Chongqing, China. Additionally, the occurrence of CDMR is related to the personal factors of pregnant women (age, parity, willingness of delivery mode) and is affected by other people (surrounding mothers, the obstetrician). Society and public media should respond with proper guidance for the adoption of cesarean section. Medical institutions should strengthen the popularization of women’s delivery knowledge during pregnancy, in particular, to alleviate the fear of vaginal delivery among older mothers and primiparous women. Obstetricians should fully understand the medical indications for cesarean delivery and offer pregnant women a full opportunity to attempt vaginal delivery. Lastly, it is recommended that health administrators execute closer and more strict supervision in terms of the introduction of cesarean section to avoid unnecessary cesarean delivery.

## Supplementary Information

**Additional file 1.** Questionnaires for the collection of basic information and follow-up data throughout the pregnancy

## Data Availability

The datasets analyzed in the current study are not publicly available due to the ongoing cohort study but are available from the corresponding author upon reasonable request.

## Abbreviations

CDMR: Cesarean delivery on maternal request
OVD: Operative vaginal delivery
