# Supplementary material for: Cesarean delivery on maternal request and its influencing factors in Chongqing, China
Source: BMC Pregnancy Childbirth. 2021 May 19;21:384. doi: 10.1186/s12884-021-03866-7 (PMC8132350; doi:10.1186/s12884-021-03866-7)
Supplement: Supplementary file 1 — Additional file 1. Questionnaires for the collection of basic information and follow-up data throughout the pregnancy [file 12884_2021_3866_MOESM1_ESM.docx]

**Additional file 1.** **Questionnaires for the collection of basic information and follow-up data throughout the pregnancy.**

Note: Subjects were administered with questionnaires in Chinese. The following English questionnaires were translated from the original Chinese version and for reference only.

**Basic information collection table for pregnant women**

Age：

Last menstrual period: / /

Hospital：

**Record of the first prenatal examination：**

Examination Time: / /

1.Whether the condition is abnormal before this pregnancy examination: No Yes

2.height(cm): weight(kg): BMI BP(mmHg):

3.Menstrual history: Last menstrual period: Expected date of childbirth:

4.Drinking history: No Yes( Alcohol consumption )

5.Smoking history:(Active Passive No) year; average per day

6. Previous Medical History: No Yes

Anemia Thyroid disease Cardiopathy Hepatic disease Hypertension Kidney disease Diabetes

Tuberculosis Hemopathy Psychosis Genital deformity Syphilis HIV Others:

Operation history and time:

7. Pregnancy and childbirth history: No Yes

Gravidity Parity (Vaginal delivery Cesarean section )

Spontaneous abortion Induced abortion Premature delivery Stillbirth

8. Family history: No Yes

Hereditary disease Developmental malformation Thyroid disease Tumor Psychosis Others:

**Early pregnancy questionnaire**

Questionnaire survey time: / /

**1.Baseline information**

1.1 Place of residence:①Urban ②Rural

1.2 Education (last grade completed):①Primary school or below ②Junior high school③Senior high /Vocational high/Technical secondary school

④Junior College/Undergraduate ⑤Postgraduate

1.3 Occupation: ①Staff and workers of government organs and institutions ②Enterprise staff ③Catering, sales and other service industries

④Self-employed person ⑤Farmers ⑥Housewives ⑦Unemployed ⑧Others:

1.4 Monthly per capita household income (RMB): ①≤1000 ②1001~3000 ③3001~5000 ④5001~10000 ⑤10001~15000 ⑥≥15001

1.5 Payment method of medical expenses: ①At one's own expense ②Basic medical insurance for urban workers (including maternity insurance) ③Basic medical insurance for urban residents (including civil affairs assistance) ④New Rural Cooperative medical Care (including civil affairs assistance) ⑤Commercial health insurance ⑥Others:

**2.** **Prenatal status of pregnant women**

2.1 Exercise during pregnancy: ①Yes ②No

2.2 Number of times received health education during pregnancy from the school for pregnant women: ①0 ②1-5 ③6-10 ④11-15 ⑤≥16

2.3 Medical staff service: ①Good ②General ③Poor

2.4 Delivery mode of surrounding mothers: ①Vaginal delivery mainly ②Cesarean section mainly ③The two delivery methods are equal

2.5 Willingness of delivery mode: (If ⑤⑥ is selected, skip 2.5.1;If ③④ is selected, skip 2.5.2)

①No consideration has been given to how to give birth at present, prenatal as advised by the doctor ②No consideration has been given to how to give birth at present,, prenatal depends on the situation ③Be sure to choose vaginal delivery ④Tending to choose vaginal delivery ⑤Tending to choose cesarean section ⑥Be sure to choose cesarean section

2.5.1 If considering vaginal delivery, your reasons are :(multiple choice)

①Natural process, no need for cesarean section, can give birth as far as possible by oneself ②It's better for baby growth and development ③Fast recovery after delivery ④Vaginal delivery is safer ⑤Lower cost ⑥Suggestions from families and friends ⑦The abdomen is more beautiful without scars ⑧Avoid scarring the uterus, which is good for the next childbirth ⑨Others:

2.5.2 If considering cesarean section, your reasons are :(multiple choice)

①Labor pain is small, afraid of vaginal labor pain ②Faster body shape recovery ③Kids are smarter and healthier ④Safer for children ⑤It is safe to the mother and saves time and effort ⑥Protection of perineal tissue, does not affect postpartum sexual life ⑦No confidence in natural childbirth, fear of failure of natural childbirth before performing cesarean section ⑧You can choose a good day ⑨Suggestions from family and friends ⑩Precious children, such as test tube baby, years of infertility ⑪Fear of pregnancy complications such as uterine rupture ⑫The doctor suggested that cesarean section was necessary ⑬Others:

2.6 Husband's advice: ①No recommendation ②Recommend vaginal delivery ③Recommend cesarean section ④No clear recommendation

2.7 Parents’ advice: ①No recommendation ②Recommend vaginal delivery ③Recommend cesarean section ④No clear recommendation

2.8 Advice from parents in law: ①No recommendation ②Recommend vaginal delivery ③Recommend cesarean section ④No clear recommendation

2.9 Friends’ advice: ①No recommendation ②Recommend vaginal delivery ③Recommend cesarean section ④No clear recommendation

**3.** **Maternal Psychological Scale**

Please check the appropriate box according to your actual situation.

| **A** | **Family adaptation partnership growth affection and resolve index (APGAR)** | **Almost always** | **Some Of The Time** | | **Hardly ever** | |  |  |
| --- | --- | --- | --- | --- | --- | --- | --- | --- |
| **A01** | I am satisfied that I can turn to my family for help when something is troubling me |  |  | |  | |  |  |
| **A02** | I am satisfied with the way my family talks over things with me and shares problems with me |  |  | |  | |  |  |
| **A03** | I am satisfied that my family accepts and supports my wishes to take on new activities or directions |  |  | |  | |  |  |
| **A04** | I am satisifed with the way my family expresses affection, and responds to my emotions, such as anger, sorrow,or love |  |  | |  | |  |  |
| **A05** | I am satisifed with the way my family and I share time together |  |  | |  | |  |  |
| **B** | **Pregnancy pressure scale (PPS)** | **No pressure** | **Low pressure** | | **Moderate pressure** | | **Severe pressure** |  |
| **B01** | It's difficult to prepare the baby's clothes |  |  | |  | |  |  |
| **B02** | It is difficult to find a satisfactory babysitter |  |  | |  | |  |  |
| **B03** | It is difficult to choose a place for confinement in childbirth |  |  | |  | |  |  |
| **B04** | It's difficult to name a child |  |  | |  | |  |  |
| **B05** | It is difficult to give the baby a physical examination |  |  | |  | |  |  |
| **B06** | Being forced to give up work for fear of having children |  |  | |  | |  |  |
| **B07** | Worried about important people not accepting children |  |  | |  | |  |  |
| **B08** | Failure to arrange chores during childbirth |  |  | |  | |  |  |
| **B09** | Fear of not getting enough psychological support |  |  | |  | |  |  |
| **B10** | It is difficult to decide how to feed the baby |  |  | |  | |  |  |
| **B11** | Worried about the sex of the baby is not as expected |  |  | |  | |  |  |
| **B12** | Affecting your sexual life |  |  | |  | |  |  |
| **B13** | Fearing that children will not be liked |  |  | |  | |  |  |
| **B14** | Worried about raising children in the future |  |  | |  | |  |  |
| **B15** | Worried about having less free time after having a baby |  |  | |  | |  |  |
| **B16** | Worried about whether the baby will be delivered safely |  |  | |  | |  |  |
| **B17** | Worried about the baby's abnormality |  |  | |  | |  |  |
| **B18** | Worried about the safety of childbirth |  |  | |  | |  |  |
| **B19** | Worried about premature |  |  | |  | |  |  |
| **B20** | Worried about the weight of the baby |  |  | |  | |  |  |
| **B21** | Worried about the possibility of abnormal delivery or cesarean section |  |  | |  | |  |  |
| **B22** | It is feared that the doctor will not arrive in time for the delivery |  |  | |  | |  |  |
| **B23** | Fear of severe pain |  |  | |  | |  |  |
| **B24** | Worried about body shape changes |  |  | |  | |  |  |
| **B25** | Worried about pregnancy spots on your face |  |  | |  | |  |  |
| **B26** | Worried about getting too fat |  |  | |  | |  |  |
| **B27** | Worried about not being able to control your clumsy body |  |  | |  | |  |  |
| **B28** | Worried about not taking care of the baby |  |  | |  | |  |  |
| **B29** | The fear of having children will affect the couple's relationship |  |  | |  | |  |  |
| **B30** | Worried about not providing good living conditions for children |  |  | |  | |  |  |
| **C** | **Hamilton anxiety scale (HAMA)** | **Not Present** | **Mild** | | **Moderate** | | **Severe** | **Very Severe** |
| **C01** | **Anxious Mood**  Worries, anticipation of the worst, fearful anticipation, irritability. |  |  | |  | |  |  |
| **C02** | **Tension**  Feelings of tension, fatigability, startle response, moved to tears easily, trembling, feelings of restlessness, inability to relax. |  |  | |  | |  |  |
| **C03** | **Fears**  Of dark, of strangers, of being left alone, of animals, of traffic, of crowds. |  |  | |  | |  |  |
| **C04** | **Insomnia**  Difficulty in falling asleep, broken sleep, unsatisfying sleep and fatigue on waking, dreams, nightmares, night terrors. |  |  | |  | |  |  |
| **C05** | **Insomnia**  Difficulty in falling asleep, broken sleep, unsatisfying sleep and fatigue on waking, dreams, nightmares, night terrors. |  |  | |  | |  |  |
| **C06** | **Depressed Mood**  Loss of interest, lack of pleasure in hobbies, depression, early waking, diurnal swing. |  |  | |  | |  |  |
| **C07** | **Somatic (muscular)**  Pains and aches, twitching, stiffness, myoclonic jerks, grinding of teeth, unsteady voice, increased muscular tone. |  |  | |  | |  |  |
| **C08** | **Somatic (sensory)**  Tinnitus, blurring of vision, hot and cold flushes, feelings of weakness, pricking sensation. |  |  | |  | |  |  |
| **C09** | **Cardiovascular Symptoms**  Tachycardia, palpitations, pain in chest, throbbing of vessels, fainting feelings, missing beat |  |  | |  | |  |  |
| **C10** | **Respiratory Symptoms**  Pressure or constriction in chest, choking feelings, sighing, dyspnea. |  |  | |  | |  |  |
| **C11** | **Gastrointestinal Symptoms**  Difficulty in swallowing, wind abdominal pain, burning sensations, abdominal fullness, nausea, vomiting, borborygmi, looseness of bowels, loss of weight, constipation. |  |  | |  | |  |  |
| **C12** | **Genitourinary Symptoms**  Frequency of micturition, urgency of micturition, amenorrhea, menorrhagia, development of rigidity, premature ejaculation, loss of libido, impotence. |  |  | |  | |  |  |
| **C13** | **Autonomic Symptoms**  Dry mouth, flushing, pallor, tendency to sweat, giddiness, tension headache, raising of hair. |  |  | |  | |  |  |
| **C14** | **Behavior at Interview**  Fidgeting, restlessness or pacing, tremor of hands, furrowed brow, strained face, sighing or rapid respiration, facial pallor, swallowing, etc. |  |  | |  | |  |  |
| **D** | **Eelf-rating depression scale(SDS) (parts)** | **A Little Of The Time** | **Some Of The Time** | | **Good Part Of The Time** | | **Most Of The Time** |  |
| **D01** | I feel down hearted and blue |  |  | |  | |  |  |
| **D02** | Morning is when I feel the best |  |  | |  | |  |  |
| **D03** | I eat as much as I used to |  |  | |  | |  |  |
| **D04** | I eat as much as I used to |  |  | |  | |  |  |
| **D05** | I find it easy to do the things I used to |  |  | |  | |  |  |
| **D06** | I feel hopeful about the future |  |  | |  | |  |  |
| **D07** | I feel hopeful about the future |  |  | |  | |  |  |
| **D08** | I feel that I am useful and needed |  |  | |  | |  |  |
| **D09** | My life is pretty full |  |  | |  | |  |  |
| **D10** | I feel that others would be better off if I were dead |  |  | |  | |  |  |
| **E** | **Social support rating scale (SSRS)** | | | | | | | |
| **E01** | **How many close friends do you have who can get support and help?** | | ①0 | | | | |  |
|  |  |  | ②1—2 | | | | |  |
|  |  |  | ③3-5 | | | | |  |
|  |  |  | ④＞6 | | | | |  |
| **E02** | **In the past year:** | | 1. Living away from family and living alone in one room | | | | |  |
|  |  |  | ②Living quarters change frequently and spend most of the time with strangers | | | | |  |
|  |  |  | 1. Living with classmates, colleagues or friends | | | | |  |
|  |  |  | ④Living with your family | | | | |  |
| **E03** | **You with your neighbors：** | | 1. You never cared about each other, you were just nodding acquaintances | | | | |  |
|  |  |  | ②You may be slightly concerned about difficulties | | | | |  |
|  |  |  | ③Some of your neighbors care about you | | | | |  |
|  |  |  | ④Most of your neighbors care a lot about you | | | | |  |
| **E04** | **You with your colleagues：** | | 1. You never cared about each other, you were just nodding acquaintances | | | | |  |
|  |  |  | 1. You may be slightly concerned about difficulties | | | | |  |
|  |  |  | 1. Some of your colleagues care about you | | | | |  |
|  |  |  | 1. Most of your colleagues care a lot about you | | | | |  |
| **E05** | **Support and care received from family members:** | | Husband (lovers) | | | ①No | |  |
|  |  |  |  |  |  | ②Seldom | |  |
|  |  |  |  |  |  | ③General | |  |
|  |  |  |  |  |  | ④Full support | |  |
|  |  |  | Parents | | | ①No | |  |
|  |  |  |  |  |  | ②Seldom | |  |
|  |  |  |  |  |  | ③General | |  |
|  |  |  |  |  |  | ④Full support | |  |
|  |  |  | Brothers and sisters | | | ①No | |  |
|  |  |  |  |  |  | ②Seldom | |  |
|  |  |  |  |  |  | ③General | |  |
|  |  |  |  |  |  | ④Full support | |  |
|  |  |  | The other members  (e.g. Sister-in-law) | | | ①No | |  |
|  |  |  |  |  |  | ②Seldom | |  |
|  |  |  |  |  |  | ③General | |  |
|  |  |  |  |  |  | ④Full support | |  |
| **E06** | **In the past, when you were in a difficult situation, the sources of financial support and help to solve practical problems were** | | （1）Without any source | | | | |  |
|  |  |  | （2）The following sources  :(more than one optional) | 1. Spouse | | | |  |
|  |  |  |  | 1. Other family members | | | |  |
|  |  |  |  | 1. Friends | | | |  |
|  |  |  |  | 1. Relatives | | | |  |
|  |  |  |  | 1. Colleagues | | | |  |
|  |  |  |  | 1. Work units | | | |  |
|  |  |  |  | 1. An official or semi-official organization such as a party, league, or trade union | | | |  |
|  |  |  |  | 1. Non-official organizations such as religious and social organizations | | | |  |
|  |  |  |  | 1. Others____________ | | | |  |
| **E07** | **The way you talk about your troubles:** | | 1. Never complained to anyone | | | | |  |
|  |  |  | 1. Speaking only to 1 or 2 individuals who are in a very close relationship | | | | |  |
|  |  |  | 1. You will tell if a friend asks | | | | |  |
|  |  |  | ④ Taking the initiative to tell your troubles to get support and understanding | | | | |  |
| **E08** | **The way to ask for help when you are in trouble:** | | ①Rely only on yourself, don't accept help from others | | | | |  |
|  |  |  | ②Seldom ask for help | | | | |  |
|  |  |  | ③Sometimes ask for help | | | | |  |
|  |  |  | ④Often ask for help from families, friends and organizations when in trouble | | | | |  |
| **E09** | **For groups (such as party and caucus organizations, religious organizations, trade unions, student unions, etc.) to organize activities, you：** | | ①Never | | | | |  |
|  |  |  | ②Sometimes | | | | |  |
|  |  |  | ③Frequently | | | | |  |
|  |  |  | ④Take the initiative to participate and be active | | | | |  |

**Investigator's signature:** ________________

**Middle pregnancy questionnaire**

Questionnaire survey time: / /

**1.** **Prenatal status of pregnant women**

1.1 Exercise during pregnancy: ①Yes ②No

1.2 Number of times received health education during pregnancy from the school for pregnant women: ①0 ②1-5 ③6-10 ④11-15 ⑤≥16

1.3 Medical staff service: ①Good ②General ③Poor

1.4 Delivery mode of surrounding mothers: ①Vaginal delivery mainly ②Cesarean section mainly ③The two delivery methods are equal

1.5 Willingness of delivery mode: (If ⑤⑥ is selected, skip 1.5.1;If ③④ is selected, skip 1.5.2)

①No consideration has been given to how to give birth at present, prenatal as advised by the doctor ②No consideration has been given to how to give birth at present,, prenatal depends on the situation ③Be sure to choose vaginal delivery ④Tending to choose vaginal delivery ⑤Tending to choose cesarean section ⑥Be sure to choose cesarean section

1.5.1 If considering vaginal delivery, your reasons are :(multiple choice)

①Natural process, no need for cesarean section, can give birth as far as possible by oneself ②It's better for baby growth and development ③Fast recovery after delivery ④Vaginal delivery is safer ⑤Lower cost ⑥Suggestions from families and friends ⑦The abdomen is more beautiful without scars ⑧Avoid scarring the uterus, which is good for the next childbirth ⑨Others:

1.5.2 If considering cesarean section, your reasons are :(multiple choice)

①Labor pain is small, afraid of vaginal labor pain ②Faster body shape recovery ③Kids are smarter and healthier ④Safer for children ⑤It is safe to the mother and saves time and effort ⑥Protection of perineal tissue, does not affect postpartum sexual life ⑦No confidence in natural childbirth, fear of failure of natural childbirth before performing cesarean section ⑧You can choose a good day ⑨Suggestions from family and friends ⑩Precious children, such as test tube baby, years of infertility ⑪Fear of pregnancy complications such as uterine rupture ⑫The doctor suggested that cesarean section was necessary ⑬Others:

1.6 Husband's advice: ①No recommendation ②Recommend vaginal delivery ③Recommend cesarean section ④No clear recommendation

1.7 Parents’ advice: ①No recommendation ②Recommend vaginal delivery ③Recommend cesarean section ④No clear recommendation

1.8 Advice from parents in law: ①No recommendation ②Recommend vaginal delivery ③Recommend cesarean section ④No clear recommendation

1.9 Friends’ advice: ①No recommendation ②Recommend vaginal delivery ③Recommend cesarean section ④No clear recommendation

**2.** **Maternal Psychological Scale**

Please check the appropriate box according to your actual situation.

| **A** | **Family adaptation partnership growth affection and resolve index (APGAR)** | **Almost always** | **Some Of The Time** | | **Hardly ever** | |  |  |
| --- | --- | --- | --- | --- | --- | --- | --- | --- |
| **A01** | I am satisfied that I can turn to my family for help when something is troubling me |  |  | |  | |  |  |
| **A02** | I am satisfied with the way my family talks over things with me and shares problems with me |  |  | |  | |  |  |
| **A03** | I am satisfied that my family accepts and supports my wishes to take on new activities or directions |  |  | |  | |  |  |
| **A04** | I am satisifed with the way my family expresses affection, and responds to my emotions, such as anger, sorrow,or love |  |  | |  | |  |  |
| **A05** | I am satisifed with the way my family and I share time together |  |  | |  | |  |  |
| **B** | **Pregnancy pressure scale (PPS)** | **No pressure** | **Low pressure** | | **Moderate pressure** | | **Severe pressure** |  |
| **B01** | It's difficult to prepare the baby's clothes |  |  | |  | |  |  |
| **B02** | It is difficult to find a satisfactory babysitter |  |  | |  | |  |  |
| **B03** | It is difficult to choose a place for confinement in childbirth |  |  | |  | |  |  |
| **B04** | It's difficult to name a child |  |  | |  | |  |  |
| **B05** | It is difficult to give the baby a physical examination |  |  | |  | |  |  |
| **B06** | Being forced to give up work for fear of having children |  |  | |  | |  |  |
| **B07** | Worried about important people not accepting children |  |  | |  | |  |  |
| **B08** | Failure to arrange chores during childbirth |  |  | |  | |  |  |
| **B09** | Fear of not getting enough psychological support |  |  | |  | |  |  |
| **B10** | It is difficult to decide how to feed the baby |  |  | |  | |  |  |
| **B11** | Worried about the sex of the baby is not as expected |  |  | |  | |  |  |
| **B12** | Affecting your sexual life |  |  | |  | |  |  |
| **B13** | Fearing that children will not be liked |  |  | |  | |  |  |
| **B14** | Worried about raising children in the future |  |  | |  | |  |  |
| **B15** | Worried about having less free time after having a baby |  |  | |  | |  |  |
| **B16** | Worried about whether the baby will be delivered safely |  |  | |  | |  |  |
| **B17** | Worried about the baby's abnormality |  |  | |  | |  |  |
| **B18** | Worried about the safety of childbirth |  |  | |  | |  |  |
| **B19** | Worried about premature |  |  | |  | |  |  |
| **B20** | Worried about the weight of the baby |  |  | |  | |  |  |
| **B21** | Worried about the possibility of abnormal delivery or cesarean section |  |  | |  | |  |  |
| **B22** | It is feared that the doctor will not arrive in time for the delivery |  |  | |  | |  |  |
| **B23** | Fear of severe pain |  |  | |  | |  |  |
| **B24** | Worried about body shape changes |  |  | |  | |  |  |
| **B25** | Worried about pregnancy spots on your face |  |  | |  | |  |  |
| **B26** | Worried about getting too fat |  |  | |  | |  |  |
| **B27** | Worried about not being able to control your clumsy body |  |  | |  | |  |  |
| **B28** | Worried about not taking care of the baby |  |  | |  | |  |  |
| **B29** | The fear of having children will affect the couple's relationship |  |  | |  | |  |  |
| **B30** | Worried about not providing good living conditions for children |  |  | |  | |  |  |
| **C** | **Hamilton anxiety scale (HAMA)** | **Not Present** | **Mild** | | **Moderate** | | **Severe** | **Very Severe** |
| **C01** | **Anxious Mood**  Worries, anticipation of the worst, fearful anticipation, irritability. |  |  | |  | |  |  |
| **C02** | **Tension**  Feelings of tension, fatigability, startle response, moved to tears easily, trembling, feelings of restlessness, inability to relax. |  |  | |  | |  |  |
| **C03** | **Fears**  Of dark, of strangers, of being left alone, of animals, of traffic, of crowds. |  |  | |  | |  |  |
| **C04** | **Insomnia**  Difficulty in falling asleep, broken sleep, unsatisfying sleep and fatigue on waking, dreams, nightmares, night terrors. |  |  | |  | |  |  |
| **C05** | **Insomnia**  Difficulty in falling asleep, broken sleep, unsatisfying sleep and fatigue on waking, dreams, nightmares, night terrors. |  |  | |  | |  |  |
| **C06** | **Depressed Mood**  Loss of interest, lack of pleasure in hobbies, depression, early waking, diurnal swing. |  |  | |  | |  |  |
| **C07** | **Somatic (muscular)**  Pains and aches, twitching, stiffness, myoclonic jerks, grinding of teeth, unsteady voice, increased muscular tone. |  |  | |  | |  |  |
| **C08** | **Somatic (sensory)**  Tinnitus, blurring of vision, hot and cold flushes, feelings of weakness, pricking sensation. |  |  | |  | |  |  |
| **C09** | **Cardiovascular Symptoms**  Tachycardia, palpitations, pain in chest, throbbing of vessels, fainting feelings, missing beat |  |  | |  | |  |  |
| **C10** | **Respiratory Symptoms**  Pressure or constriction in chest, choking feelings, sighing, dyspnea. |  |  | |  | |  |  |
| **C11** | **Gastrointestinal Symptoms**  Difficulty in swallowing, wind abdominal pain, burning sensations, abdominal fullness, nausea, vomiting, borborygmi, looseness of bowels, loss of weight, constipation. |  |  | |  | |  |  |
| **C12** | **Genitourinary Symptoms**  Frequency of micturition, urgency of micturition, amenorrhea, menorrhagia, development of rigidity, premature ejaculation, loss of libido, impotence. |  |  | |  | |  |  |
| **C13** | **Autonomic Symptoms**  Dry mouth, flushing, pallor, tendency to sweat, giddiness, tension headache, raising of hair. |  |  | |  | |  |  |
| **C14** | **Behavior at Interview**  Fidgeting, restlessness or pacing, tremor of hands, furrowed brow, strained face, sighing or rapid respiration, facial pallor, swallowing, etc. |  |  | |  | |  |  |
| **D** | **Eelf-rating depression scale(SDS) (parts)** | **A Little Of The Time** | **Some Of The Time** | | **Good Part Of The Time** | | **Most Of The Time** |  |
| **D01** | I feel down hearted and blue |  |  | |  | |  |  |
| **D02** | Morning is when I feel the best |  |  | |  | |  |  |
| **D03** | I eat as much as I used to |  |  | |  | |  |  |
| **D04** | I eat as much as I used to |  |  | |  | |  |  |
| **D05** | I find it easy to do the things I used to |  |  | |  | |  |  |
| **D06** | I feel hopeful about the future |  |  | |  | |  |  |
| **D07** | I feel hopeful about the future |  |  | |  | |  |  |
| **D08** | I feel that I am useful and needed |  |  | |  | |  |  |
| **D09** | My life is pretty full |  |  | |  | |  |  |
| **D10** | I feel that others would be better off if I were dead |  |  | |  | |  |  |
| **E** | **Social support rating scale (SSRS)** | | | | | | | |
| **E01** | **How many close friends do you have who can get support and help?** | | ①0 | | | | |  |
|  |  |  | ②1—2 | | | | |  |
|  |  |  | ③3-5 | | | | |  |
|  |  |  | ④＞6 | | | | |  |
| **E02** | **In the past year:** | | 1. Living away from family and living alone in one room | | | | |  |
|  |  |  | ②Living quarters change frequently and spend most of the time with strangers | | | | |  |
|  |  |  | 1. Living with classmates, colleagues or friends | | | | |  |
|  |  |  | ④Living with your family | | | | |  |
| **E03** | **You with your neighbors：** | | 1. You never cared about each other, you were just nodding acquaintances | | | | |  |
|  |  |  | ②You may be slightly concerned about difficulties | | | | |  |
|  |  |  | ③Some of your neighbors care about you | | | | |  |
|  |  |  | ④Most of your neighbors care a lot about you | | | | |  |
| **E04** | **You with your colleagues：** | | 1. You never cared about each other, you were just nodding acquaintances | | | | |  |
|  |  |  | 1. You may be slightly concerned about difficulties | | | | |  |
|  |  |  | 1. Some of your colleagues care about you | | | | |  |
|  |  |  | 1. Most of your colleagues care a lot about you | | | | |  |
| **E05** | **Support and care received from family members:** | | Husband (lovers) | | | ①No | |  |
|  |  |  |  |  |  | ②Seldom | |  |
|  |  |  |  |  |  | ③General | |  |
|  |  |  |  |  |  | ④Full support | |  |
|  |  |  | Parents | | | ①No | |  |
|  |  |  |  |  |  | ②Seldom | |  |
|  |  |  |  |  |  | ③General | |  |
|  |  |  |  |  |  | ④Full support | |  |
|  |  |  | Brothers and sisters | | | ①No | |  |
|  |  |  |  |  |  | ②Seldom | |  |
|  |  |  |  |  |  | ③General | |  |
|  |  |  |  |  |  | ④Full support | |  |
|  |  |  | The other members  (e.g. Sister-in-law) | | | ①No | |  |
|  |  |  |  |  |  | ②Seldom | |  |
|  |  |  |  |  |  | ③General | |  |
|  |  |  |  |  |  | ④Full support | |  |
| **E06** | **In the past, when you were in a difficult situation, the sources of financial support and help to solve practical problems were** | | （1）Without any source | | | | |  |
|  |  |  | （2）The following sources  :(more than one optional) | 1. Spouse | | | |  |
|  |  |  |  | 1. Other family members | | | |  |
|  |  |  |  | 1. Friends | | | |  |
|  |  |  |  | 1. Relatives | | | |  |
|  |  |  |  | 1. Colleagues | | | |  |
|  |  |  |  | 1. Work units | | | |  |
|  |  |  |  | 1. An official or semi-official organization such as a party, league, or trade union | | | |  |
|  |  |  |  | 1. Non-official organizations such as religious and social organizations | | | |  |
|  |  |  |  | 1. Others____________ | | | |  |
| **E07** | **The way you talk about your troubles:** | | 1. Never complained to anyone | | | | |  |
|  |  |  | 1. Speaking only to 1 or 2 individuals who are in a very close relationship | | | | |  |
|  |  |  | 1. You will tell if a friend asks | | | | |  |
|  |  |  | ④ Taking the initiative to tell your troubles to get support and understanding | | | | |  |
| **E08** | **The way to ask for help when you are in trouble:** | | ①Rely only on yourself, don't accept help from others | | | | |  |
|  |  |  | ②Seldom ask for help | | | | |  |
|  |  |  | ③Sometimes ask for help | | | | |  |
|  |  |  | ④Often ask for help from families, friends and organizations when in trouble | | | | |  |
| **E09** | **For groups (such as party and caucus organizations, religious organizations, trade unions, student unions, etc.) to organize activities, you：** | | ①Never | | | | |  |
|  |  |  | ②Sometimes | | | | |  |
|  |  |  | ③Frequently | | | | |  |
|  |  |  | ④Take the initiative to participate and be active | | | | |  |

**Investigator's signature:** ________________

**Late pregnancy questionnaire**

Questionnaire survey time: / /

**1.** **Prenatal status of pregnant women**

1.1 Exercise during pregnancy: ①Yes ②No

1.2 Number of times received health education during pregnancy from the school for pregnant women: ①0 ②1-5 ③6-10 ④11-15 ⑤≥16

1.3 Times of prenatal inspection: ①＜6 ②6-10 ③11-15 ④＞15

1.4 There were abnormalities in the prenatal inspection: ①Yes ②No

1.5 Medical staff service: ①Good ②General ③Poor

1.6 Delivery mode of surrounding mothers: ①Vaginal delivery mainly ②Cesarean section mainly ③The two delivery methods are equal

1.7 Willingness of delivery mode: (If ⑤⑥ is selected, skip 1.7.1;If ③④ is selected, skip 1.7.2)

①No consideration has been given to how to give birth at present, prenatal as advised by the doctor ②No consideration has been given to how to give birth at present,, prenatal depends on the situation ③Be sure to choose vaginal delivery ④Tending to choose vaginal delivery ⑤Tending to choose cesarean section ⑥Be sure to choose cesarean section

1.7.1 If considering vaginal delivery, your reasons are :(multiple choice)

①Natural process, no need for cesarean section, can give birth as far as possible by oneself ②It's better for baby growth and development ③Fast recovery after delivery ④Vaginal delivery is safer ⑤Lower cost ⑥Suggestions from families and friends ⑦The abdomen is more beautiful without scars ⑧Avoid scarring the uterus, which is good for the next childbirth ⑨Others:

1.7.2 If considering cesarean section, your reasons are :(multiple choice)

①Labor pain is small, afraid of vaginal labor pain ②Faster body shape recovery ③Kids are smarter and healthier ④Safer for children ⑤It is safe to the mother and saves time and effort ⑥Protection of perineal tissue, does not affect postpartum sexual life ⑦No confidence in natural childbirth, fear of failure of natural childbirth before performing cesarean section ⑧You can choose a good day ⑨Suggestions from family and friends ⑩Precious children, such as test tube baby, years of infertility ⑪Fear of pregnancy complications such as uterine rupture ⑫The doctor suggested that cesarean section was necessary ⑬Others:

1.8 Husband's advice: ①No recommendation ②Recommend vaginal delivery ③Recommend cesarean section ④No clear recommendation

1.9 Parents’ advice: ①No recommendation ②Recommend vaginal delivery ③Recommend cesarean section ④No clear recommendation

1.10 Advice from parents in law: ①No recommendation ②Recommend vaginal delivery ③Recommend cesarean section ④No clear recommendation

1.11 Friends’ advice: ①No recommendation ②Recommend vaginal delivery ③Recommend cesarean section ④No clear recommendation

1.12 Doctor's advice: ①No recommendation ②Recommend vaginal delivery ③Recommend cesarean section ④No clear recommendation

**2.** **Maternal Psychological Scale**

Please check the appropriate box according to your actual situation.

| **A** | **Family adaptation partnership growth affection and resolve index (APGAR)** | **Almost always** | **Some Of The Time** | | **Hardly ever** | |  |  |
| --- | --- | --- | --- | --- | --- | --- | --- | --- |
| **A01** | I am satisfied that I can turn to my family for help when something is troubling me |  |  | |  | |  |  |
| **A02** | I am satisfied with the way my family talks over things with me and shares problems with me |  |  | |  | |  |  |
| **A03** | I am satisfied that my family accepts and supports my wishes to take on new activities or directions |  |  | |  | |  |  |
| **A04** | I am satisifed with the way my family expresses affection, and responds to my emotions, such as anger, sorrow,or love |  |  | |  | |  |  |
| **A05** | I am satisifed with the way my family and I share time together |  |  | |  | |  |  |
| **B** | **Pregnancy pressure scale (PPS)** | **No pressure** | **Low pressure** | | **Moderate pressure** | | **Severe pressure** |  |
| **B01** | It's difficult to prepare the baby's clothes |  |  | |  | |  |  |
| **B02** | It is difficult to find a satisfactory babysitter |  |  | |  | |  |  |
| **B03** | It is difficult to choose a place for confinement in childbirth |  |  | |  | |  |  |
| **B04** | It's difficult to name a child |  |  | |  | |  |  |
| **B05** | It is difficult to give the baby a physical examination |  |  | |  | |  |  |
| **B06** | Being forced to give up work for fear of having children |  |  | |  | |  |  |
| **B07** | Worried about important people not accepting children |  |  | |  | |  |  |
| **B08** | Failure to arrange chores during childbirth |  |  | |  | |  |  |
| **B09** | Fear of not getting enough psychological support |  |  | |  | |  |  |
| **B10** | It is difficult to decide how to feed the baby |  |  | |  | |  |  |
| **B11** | Worried about the sex of the baby is not as expected |  |  | |  | |  |  |
| **B12** | Affecting your sexual life |  |  | |  | |  |  |
| **B13** | Fearing that children will not be liked |  |  | |  | |  |  |
| **B14** | Worried about raising children in the future |  |  | |  | |  |  |
| **B15** | Worried about having less free time after having a baby |  |  | |  | |  |  |
| **B16** | Worried about whether the baby will be delivered safely |  |  | |  | |  |  |
| **B17** | Worried about the baby's abnormality |  |  | |  | |  |  |
| **B18** | Worried about the safety of childbirth |  |  | |  | |  |  |
| **B19** | Worried about premature |  |  | |  | |  |  |
| **B20** | Worried about the weight of the baby |  |  | |  | |  |  |
| **B21** | Worried about the possibility of abnormal delivery or cesarean section |  |  | |  | |  |  |
| **B22** | It is feared that the doctor will not arrive in time for the delivery |  |  | |  | |  |  |
| **B23** | Fear of severe pain |  |  | |  | |  |  |
| **B24** | Worried about body shape changes |  |  | |  | |  |  |
| **B25** | Worried about pregnancy spots on your face |  |  | |  | |  |  |
| **B26** | Worried about getting too fat |  |  | |  | |  |  |
| **B27** | Worried about not being able to control your clumsy body |  |  | |  | |  |  |
| **B28** | Worried about not taking care of the baby |  |  | |  | |  |  |
| **B29** | The fear of having children will affect the couple's relationship |  |  | |  | |  |  |
| **B30** | Worried about not providing good living conditions for children |  |  | |  | |  |  |
| **C** | **Hamilton anxiety scale (HAMA)** | **Not Present** | **Mild** | | **Moderate** | | **Severe** | **Very Severe** |
| **C01** | **Anxious Mood**  Worries, anticipation of the worst, fearful anticipation, irritability. |  |  | |  | |  |  |
| **C02** | **Tension**  Feelings of tension, fatigability, startle response, moved to tears easily, trembling, feelings of restlessness, inability to relax. |  |  | |  | |  |  |
| **C03** | **Fears**  Of dark, of strangers, of being left alone, of animals, of traffic, of crowds. |  |  | |  | |  |  |
| **C04** | **Insomnia**  Difficulty in falling asleep, broken sleep, unsatisfying sleep and fatigue on waking, dreams, nightmares, night terrors. |  |  | |  | |  |  |
| **C05** | **Insomnia**  Difficulty in falling asleep, broken sleep, unsatisfying sleep and fatigue on waking, dreams, nightmares, night terrors. |  |  | |  | |  |  |
| **C06** | **Depressed Mood**  Loss of interest, lack of pleasure in hobbies, depression, early waking, diurnal swing. |  |  | |  | |  |  |
| **C07** | **Somatic (muscular)**  Pains and aches, twitching, stiffness, myoclonic jerks, grinding of teeth, unsteady voice, increased muscular tone. |  |  | |  | |  |  |
| **C08** | **Somatic (sensory)**  Tinnitus, blurring of vision, hot and cold flushes, feelings of weakness, pricking sensation. |  |  | |  | |  |  |
| **C09** | **Cardiovascular Symptoms**  Tachycardia, palpitations, pain in chest, throbbing of vessels, fainting feelings, missing beat |  |  | |  | |  |  |
| **C10** | **Respiratory Symptoms**  Pressure or constriction in chest, choking feelings, sighing, dyspnea. |  |  | |  | |  |  |
| **C11** | **Gastrointestinal Symptoms**  Difficulty in swallowing, wind abdominal pain, burning sensations, abdominal fullness, nausea, vomiting, borborygmi, looseness of bowels, loss of weight, constipation. |  |  | |  | |  |  |
| **C12** | **Genitourinary Symptoms**  Frequency of micturition, urgency of micturition, amenorrhea, menorrhagia, development of rigidity, premature ejaculation, loss of libido, impotence. |  |  | |  | |  |  |
| **C13** | **Autonomic Symptoms**  Dry mouth, flushing, pallor, tendency to sweat, giddiness, tension headache, raising of hair. |  |  | |  | |  |  |
| **C14** | **Behavior at Interview**  Fidgeting, restlessness or pacing, tremor of hands, furrowed brow, strained face, sighing or rapid respiration, facial pallor, swallowing, etc. |  |  | |  | |  |  |
| **D** | **Eelf-rating depression scale(SDS) (parts)** | **A Little Of The Time** | **Some Of The Time** | | **Good Part Of The Time** | | **Most Of The Time** |  |
| **D01** | I feel down hearted and blue |  |  | |  | |  |  |
| **D02** | Morning is when I feel the best |  |  | |  | |  |  |
| **D03** | I eat as much as I used to |  |  | |  | |  |  |
| **D04** | I eat as much as I used to |  |  | |  | |  |  |
| **D05** | I find it easy to do the things I used to |  |  | |  | |  |  |
| **D06** | I feel hopeful about the future |  |  | |  | |  |  |
| **D07** | I feel hopeful about the future |  |  | |  | |  |  |
| **D08** | I feel that I am useful and needed |  |  | |  | |  |  |
| **D09** | My life is pretty full |  |  | |  | |  |  |
| **D10** | I feel that others would be better off if I were dead |  |  | |  | |  |  |
| **E** | **Social support rating scale (SSRS)** | | | | | | | |
| **E01** | **How many close friends do you have who can get support and help?** | | ①0 | | | | |  |
|  |  |  | ②1—2 | | | | |  |
|  |  |  | ③3-5 | | | | |  |
|  |  |  | ④＞6 | | | | |  |
| **E02** | **In the past year:** | | 1. Living away from family and living alone in one room | | | | |  |
|  |  |  | ②Living quarters change frequently and spend most of the time with strangers | | | | |  |
|  |  |  | 1. Living with classmates, colleagues or friends | | | | |  |
|  |  |  | ④Living with your family | | | | |  |
| **E03** | **You with your neighbors：** | | 1. You never cared about each other, you were just nodding acquaintances | | | | |  |
|  |  |  | ②You may be slightly concerned about difficulties | | | | |  |
|  |  |  | ③Some of your neighbors care about you | | | | |  |
|  |  |  | ④Most of your neighbors care a lot about you | | | | |  |
| **E04** | **You with your colleagues：** | | 1. You never cared about each other, you were just nodding acquaintances | | | | |  |
|  |  |  | 1. You may be slightly concerned about difficulties | | | | |  |
|  |  |  | 1. Some of your colleagues care about you | | | | |  |
|  |  |  | 1. Most of your colleagues care a lot about you | | | | |  |
| **E05** | **Support and care received from family members:** | | Husband (lovers) | | | ①No | |  |
|  |  |  |  |  |  | ②Seldom | |  |
|  |  |  |  |  |  | ③General | |  |
|  |  |  |  |  |  | ④Full support | |  |
|  |  |  | Parents | | | ①No | |  |
|  |  |  |  |  |  | ②Seldom | |  |
|  |  |  |  |  |  | ③General | |  |
|  |  |  |  |  |  | ④Full support | |  |
|  |  |  | Brothers and sisters | | | ①No | |  |
|  |  |  |  |  |  | ②Seldom | |  |
|  |  |  |  |  |  | ③General | |  |
|  |  |  |  |  |  | ④Full support | |  |
|  |  |  | The other members  (e.g. Sister-in-law) | | | ①No | |  |
|  |  |  |  |  |  | ②Seldom | |  |
|  |  |  |  |  |  | ③General | |  |
|  |  |  |  |  |  | ④Full support | |  |
| **E06** | **In the past, when you were in a difficult situation, the sources of financial support and help to solve practical problems were** | | （1）Without any source | | | | |  |
|  |  |  | （2）The following sources  :(more than one optional) | 1. Spouse | | | |  |
|  |  |  |  | 1. Other family members | | | |  |
|  |  |  |  | 1. Friends | | | |  |
|  |  |  |  | 1. Relatives | | | |  |
|  |  |  |  | 1. Colleagues | | | |  |
|  |  |  |  | 1. Work units | | | |  |
|  |  |  |  | 1. An official or semi-official organization such as a party, league, or trade union | | | |  |
|  |  |  |  | 1. Non-official organizations such as religious and social organizations | | | |  |
|  |  |  |  | 1. Others____________ | | | |  |
| **E07** | **The way you talk about your troubles:** | | 1. Never complained to anyone | | | | |  |
|  |  |  | 1. Speaking only to 1 or 2 individuals who are in a very close relationship | | | | |  |
|  |  |  | 1. You will tell if a friend asks | | | | |  |
|  |  |  | ④ Taking the initiative to tell your troubles to get support and understanding | | | | |  |
| **E08** | **The way to ask for help when you are in trouble:** | | ①Rely only on yourself, don't accept help from others | | | | |  |
|  |  |  | ②Seldom ask for help | | | | |  |
|  |  |  | ③Sometimes ask for help | | | | |  |
|  |  |  | ④Often ask for help from families, friends and organizations when in trouble | | | | |  |
| **E09** | **For groups (such as party and caucus organizations, religious organizations, trade unions, student unions, etc.) to organize activities, you：** | | ①Never | | | | |  |
|  |  |  | ②Sometimes | | | | |  |
|  |  |  | ③Frequently | | | | |  |
|  |  |  | ④Take the initiative to participate and be active | | | | |  |

**Investigator's signature:** _______________
